# Characterisation of worldwide *Helicobacter pylori* strains reveals genetic conservation and essentiality of serine protease HtrA

**DOI:** 10.1111/mmi.13276

**Published:** 2015-12-22

**Authors:** Nicole Tegtmeyer, Yoshan Moodley, Yoshio Yamaoka, Sandy Ramona Pernitzsch, Vanessa Schmidt, Francisco Rivas Traverso, Thomas P. Schmidt, Roland Rad, Khay Guan Yeoh, Ho Bow, Javier Torres, Markus Gerhard, Gisbert Schneider, Silja Wessler, Steffen Backert

**Affiliations:** ^1^ Lehrstuhl für MikrobiologieFriedrich‐Alexander‐Universität Erlangen‐NürnbergStaudtstr. 5D‐91058ErlangenGermany; ^2^ Institut für Medizinische MikrobiologieOtto‐von‐Guericke Universität MagdeburgLeipziger Str. 44D‐39120MagdeburgGermany; ^3^ Department of ZoologyUniversity of VendaPrivate Bag X5050Thohoyandou0950South Africa; ^4^ Konrad‐Lorenz‐Institut für Vergleichende Verhaltensforschung, Department für Integrative Biologie und EvolutionVeterinärmedizinische Universität WienSavoyenstr. 1aA‐1160WienAustria; ^5^ Michael E. DeBakey Veterans Affairs Medical Center and Baylor College of MedicineDept. Medicine‐GastroenterologyHoustonTXUSA; ^6^ Oita University Faculty of MedicineDept. Environmental and Preventive MedicineYufuJapan; ^7^ Research Center for Infectious Diseases (ZINF)University of WürzburgJosef‐Schneider‐Str. 2/Bau D15D‐97080WürzburgGermany; ^8^ Department of Molecular Biology, Division of MicrobiologyParis‐Lodron University of SalzburgBillroth Str. 11A‐5020SalzburgAustria; ^9^ II Medical Department, Klinikum Rechts der IsarTechnical University MunichMunichGermany; ^10^ German Cancer Consortium (DKTK)German Cancer Research Center (DKFZ)HeidelbergGermany; ^11^ Department of Microbiology, Yong Loo Lin School of MedicineNational University of SingaporeSingapore; ^12^ Unidad de Investigacion en Enfermedades InfecciosasUMAE Pediatria, IMSSMexico CityMexico; ^13^ Institut für Medizinische Mikrobiologie, Immunologie und HygieneTechnische Universität MünchenMunich81675Germany; ^14^ ETH ZürichInstitut für Pharmazeutische WissenschaftenVladimir‐Prelog‐Weg 4CH‐8093ZürichSwitzerland

## Abstract

HtrA proteases and chaperones exhibit important roles in periplasmic protein quality control and stress responses. The genetic inactivation of htrA has been described for many bacterial pathogens. However, in some cases such as the gastric pathogen *H* *elicobacter pylori*, HtrA is secreted where it cleaves the tumour‐suppressor E‐cadherin interfering with gastric disease development, but the generation of htrA mutants is still lacking. Here, we show that the htrA gene locus is highly conserved in worldwide strains. HtrA presence was confirmed in 992 *H* *. pylori* isolates in gastric biopsy material from infected patients. Differential RNA‐sequencing (dRNA‐seq) indicated that htrA is encoded in an operon with two subsequent genes, HP1020 and HP1021. Genetic mutagenesis and complementation studies revealed that HP1020 and HP1021, but not htrA, can be mutated. In addition, we demonstrate that suppression of HtrA proteolytic activity with a newly developed inhibitor is sufficient to effectively kill *H* *. pylori*, but not other bacteria. We show that *H* *elicobacter* htrA is an essential bifunctional gene with crucial intracellular and extracellular functions. Thus, we describe here the first microbe in which htrA is an indispensable gene, a situation unique in the bacterial kingdom. HtrA can therefore be considered a promising new target for anti‐bacterial therapy.

## Introduction

When modern humans migrated out of Africa about 60,000 years ago, they were already carrying *Helicobacter pylori* in the stomachs, and these bacteria have subsequently diversified in parallel with their host (Moodley *et al*., 2012). To date, seven major *H. pylori* populations, specific to Africa (3), Asia (2), Sahul (1) and Europe (1) have been described (Falush *et al*., 2003; Linz *et al*., 2007; Moodley *et al*., 2009). During this long co‐evolutionary association, *H. pylori* developed various mechanisms to avoid clearance by the human immune system and became a paradigm to study persistent infections (Salama *et al*., 2013). Today, *H. pylori* colonises about half of the world's population, and these infections are associated with chronic, often asymptomatic gastritis in all infected individuals, while less often more severe gastric diseases, such as peptic ulceration, mucosa‐associated lymphoid tissue lymphoma and gastric adenocarcinoma, can arise (Polk and Peek, 2010). The clinical outcome of *H. pylori* infections is proposed to be dependent on complex host–pathogen interactions. Disease development is controlled by various key factors including the genetic predisposition of the host, the bacterial genotype and environmental parameters (Amieva and El‐Omar, 2008). *H. pylori* strains are highly diverse in both their genetic polymorphisms and potential to induce pathogenicity. *H. pylori* genes encoding pathogenicity‐associated and other factors rapidly evolve through mutation and recombination, changing the bacteria–host interaction (Atherton and Blaser, 2009). A multitude of bacterial factors have been reported that can influence *H. pylori* virulence. There are two major virulence determinants: the CagA protein encoded by the cytotoxin‐associated genes pathogenicity island (*cag*PAI) and the vacuolating cytotoxin (VacA). Although the *cag*PAI encodes a type IV secretion system (T4SS) for delivery of CagA into the host cell, VacA is secreted in the environment and triggers various responses such as pore formation in the membrane, modification of endo‐lysosomal trafficking, cellular vacuolation, apoptosis and immune cell inhibition (Pachathundikandi *et al*., 2013; Posselt *et al*., 2013). Other well‐known pathogenicity‐associated processes include urease‐triggered neutralisation of acidic pH, flagella‐mediated motility, expression of multiple adhesins (AlpA/B, BabA/B, HopQ, HopZ, OipA, SabA and others), inhibition of T cell proliferation by gamma‐glutamyltranspeptidase as well as secretion of the proteases such as high temperature requirement A (HtrA) (Backert *et al*., 2011; Salama *et al*., 2013).

HtrA proteins and their orthologues represent a class of evolutionarily conserved heat shock‐induced serine proteases and chaperones, which are widely distributed among prokaryotic and eukaryotic organisms including humans (Gottesman *et al*., 1997; Ingmer and Brøndsted, 2009; Frees *et al*., 2013). Although HtrA orthologues normally exhibit proteolytic activities towards many substrates, their physiological roles and architectural structures are rather diverse among different species. In many bacteria, HtrA proteases contain an amino‐terminal signal peptide, followed by a trypsin‐like serine protease module and one or two PDZ domains to enable protein–protein interactions (Kim and Kim, 2005; Backert *et al*., 2013; Skorko‐Glonek *et al*., 2013). These proteases are commonly located in the periplasmic space, where they assemble proteolytic active oligomers with a crucial function in controlling the quality of proteins (Clausen *et al*., 2002; 2011). The best characterised HtrA model systems are described for *Escherichia coli*. The *E. coli* genome encodes three HtrA members (DegP, DegQ and DegS) that exhibit differentially regulated activities. DegP is well characterised as an ATP‐independent chaperone protease, which is converted into active oligomeric forms by substrate binding and degrades unfolded proteins into small peptides (Jiang *et al*., 2008; Krojer *et al*., 2008). The regulatory protease DegS processes the anti‐sigma factor RseA, whereas the physiological functions of DegQ remain poorly understood (Bass *et al*., 1996). Each of these three HtrAs display a high degree of homology in their protease domains, and DegP and DegQ contain two PDZ domains, whereas DegS harbours only one such domain (Kim and Kim, 2005). However, DegP is a serine protease whose primary role is the digestion of abnormal proteins within the periplasmic space, and a number of proteins have been described as natural substrates (Kim and Kim, 2005; Clausen *et al*., 2011). In addition to this proteolytic activity, DegP has also been reported to exhibit chaperone activity (Spiess *et al*., 1999) at low temperatures, whereas its proteolytic activity increases rapidly at temperatures of 32–42°C (Skorko‐Glonek *et al*., 1995; Spiess *et al*., 1999). Loss of *htr*A by mutation causes high temperature sensitivity in all bacteria examined thus far (Lipinska *et al*., 1989; Pedersen *et al*., 2001; Cortés *et al*., 2002; Mo *et al*., 2006; Flannagan *et al*., 2007; Boehm *et al*., 2015).

Traditionally, it was believed that HtrA family members were operating strictly intracellularly within the periplasm of many bacteria. However, we have recently unravelled a new feature of HtrA during infection. In *H. pylori* and its close relative *Campylobacter jejuni*, HtrA proteins are actively secreted in the extracellular space, where they are able to hijack host cell proteins (Hoy *et al*., 2010; 2012; Boehm *et al*., 2012). *In vitro* infection experiments indicated that HtrA can open the cell‐to‐cell junctions in polarised cell monolayers by cleaving‐off the extracellular domain of the surface adhesion protein E‐cadherin, followed by paracellular transmigration of the bacteria (Hoy *et al*., 2010; Boehm *et al*., 2012). Deletion of the *htrA* gene in *C. jejuni* leads to a defect in E‐cadherin shedding and impaired transmigration of the bacteria across monolayers of polarised epithelial cells *in vitro* (Hoy *et al*., 2010; Boehm *et al*., 2012). In addition, using Δ*htrA* deletion mutants, it was shown that HtrA plays a pivotal role in *C. jejuni* infection, inducing host cell apoptosis and immunopathology in the mouse gut (Heimesaat *et al*., 2014a, 2014b). Likewise, HtrA is crucial for the virulence of *Chlamydia trachomatis* (Gloeckl *et al*., 2013), *Klebsiella pneumoniae* (Cortés *et al*., 2002), *Listeria monocytogenes* (Wilson *et al*., 2006), *Salmonella enterica* (Humphreys *et al*., 1999), *Shigella flexneri* (Purdy *et al*., 2002) and *Yersinia enterocolitica* (Li *et al*., 1996). In contrast, a mutant of the *htrA* gene in *H. pylori* has not yet been reported. Instead, the generation of *htrA* knockout mutants failed for hpEurope strains G27, 26695 and P12 (Salama *et al*., 2004; Hoy *et al*., 2010). It is also unknown whether populations other than hpEurope feature *htrA* in their genomes, as this would allow an understanding of that gene's evolutionary importance to *H. pylori*. Therefore, evidence that *htrA* may be an essential gene in *H. pylori* is still lacking. In addition, secretion of HtrA and E‐cadherin cleavage was, thus far, only investigated in *H. pylori* strains 26695 and P12 (Hoy *et al*., 2010). Therefore, it is not yet clear if HtrA is encoded by *H. pylori* strains worldwide, or only among European isolates. The goal of the present study was to investigate the genetic organisation of *htrA* and flanking genes, to study *htrA* transcription, to screen a wide range of worldwide strains for functional HtrA proteins and to either generate *htrA* knockout mutants or provide direct evidence in case inactivation of the gene is lethal to *H. pylori*.

## Results

### Presence of htrA gene in clinical *H* *. pylori* strains from four continents

To obtain an overview of whether the *htrA* gene is present in non‐hpEurope strains, we screened a large number of *H. pylori* strains via PCR. DNA was extracted either from *H. pylori*‐positive gastric biopsies or live *H. pylori* isolated from a large collection of 992 patients with different clinical outcome from Germany, Japan, Singapore, USA, Mexico and Colombia (Table 1). Initially, various primers were designed, covering full‐length and different fragments of the *htrA* gene. The *htrA*‐positive genotype was detected in all 992 *H. pylori* isolates (Table 1, Fig. S1A–C in the supporting information). As control, the integrity and identity of DNA was confirmed by species‐specific 16SrRNA gene PCR, yielding clear signals from all these isolates, indicating that *H. pylori* bacteria are present (data not shown). Taken together, the above data suggest that *htrA* is a highly conserved gene in *H. pylori*, present in a very high number (if not all) of isolates from Europe, Asia, North America and South America.

**Table 1:** HtrA gene presence in *H* *. pylori* of gastric biopsies from 992 patients with different clinical outcome and geographic origin

	Germany	Singapore	Mexico	USA	Japan	Columbia
Total number of strains	187	80	203	201	152	169
HtrA‐positive strains	187	80	203	201	152	169
Disease group						
Non‐atrophic gastritis	0	0	90	0	0	0
Atrophic gastritis	0	0	0	0	41	0
Gastritis	0	12	0	66	0	39
Intestinal metaplasia	0	0	20	0	0	0
Gastric cancer	0	2	49	4	18	33
Gastric ulcer	0	10	0	31	43	0
Duodenal ulcer	0	26	19	74	48	21
Gastric and duodenal ulcer	0	7	0	9	0	0
Non‐ulcer‐dyspepsia	0	14	0	0	0	0
MALT lymphoma	0	0	0	3	0	4
Lymphoma	0	0	0	9	0	0
Non‐symptomatic	0	9	0	0	0	0
Not defined	187	0	25	5	2	72

### Genetic organisation of the htrA gene region is highly conserved in all sequenced *H* *. pylori* strains

**Figure 1:**
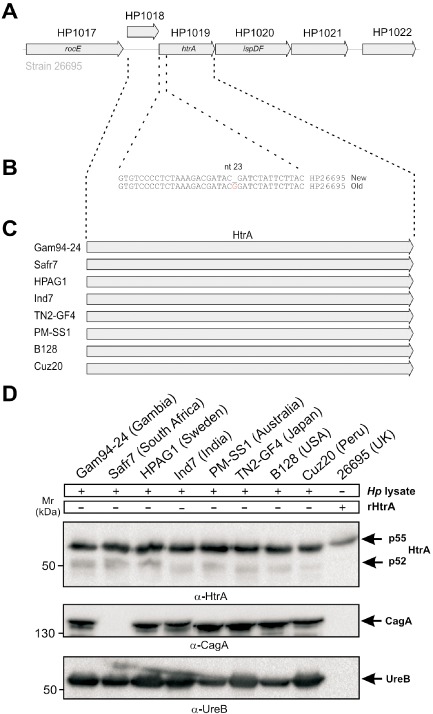
Genetic organisation of the htrA locus and HtrA expression in worldwide *H* *. pylori* strains. A. Schematic representation of the htrA gene locus in *H* *. pylori* strain 26695 (Tomb *et al*., 1997). Shown is the organisation of genes designated HP1017 to HP1022. In the original publication, the htrA gene is separated into two genes, called HP1018 and HP1019 (Tomb *et al*., 1997). The proposed start of gene HP1018 is at position 1,081,440 and HP1019 at position 1,081,537 in the 26695 genome. B. Re‐sequencing of the htrA gene locus in the same strain (G1JZF0 – G1JZF0_HELPY) has then shown that htrA is a single gene of 1,428 bp length due to a missing guanidine residue at position 1,081,558 which resulted in a frameshift and two putative genes (Löwer *et al*., 2008). C. Analysis of fully sequenced *H* *. pylori* isolates from all continents showed that the full‐length htrA gene is 1,428 bp long. D. Western blots of the HtrA proteins in these strains as well as recombinant HtrA (rHtrA) revealed bands of 52 kDa (p52) and 55 kDa (p55) as indicated by arrows. Loading control blots for CagA and urease B are shown. One of the strains, Safr7, is *cag* PAI‐negative and therefore lacks CagA.

In the first published *H. pylori* genome sequence of strain 26695, the *htrA* locus comprises 6,288 bp and contained six genes/domains (HP1017 to HP1022) organised in tight linkage (Fig. 1A). In this scheme, the *htrA* gene is separated into two genes, called HP1018 and HP1019 (Tomb *et al*., 1997). However, re‐sequencing of the *htrA* gene locus in strain 26695 suggested that the originally published *htrA* sequence possessed a potentially incorrect guanidine residue at position 1,081,558 (Fig. 1B) and, thus *htrA* is instead encoded by a single gene 1,428 bp in length (Löwer *et al*., 2008). Currently, there are 72 fully sequenced genomes available from worldwide *H. pylori* isolates (http://www.ncbi.nlm.nih.gov/genome/genomes/169). All investigated fully sequenced *H. pylori* strains contain the full‐length *htrA* gene of 1,428 bp and expressed HtrA protein species of 52 kDa (p52) and 55 kDa (p55) on Western blots (Fig. 1C and D, Table S1 in the supporting information). Their protein sequences and domain structure with signal peptide, protease module and two PDZ domains are highly conserved (Fig. S2 in the supporting information).

### Evolutionary conservation and genetic diversity and structure of the htrA region in *H* *. pylori*

In strains from Africa, Europe, Asia, Australia, North America and South America, the entire *htrA* gene region was conserved in genetic organisation and gene order but was highly variable at the nucleotide level, containing 1,296 polymorphic sites, a haplotype diversity of 0.999 and nucleotide diversity of 0.05 (Table 2). Diversity within each gene/domain was very similar, varying between 0.996–0.997 and 0.04–0.05 for haplotype and nucleotide diversity respectively. These values were also very similar to those calculated for the global data set of 769 worldwide *H. pylori* isolates (Linz *et al*., 2007). Tajima's D statistic was originally intended to detect departures from neutrality (Tajima, 1989); however, in essential housekeeping genes such as those used in the multi‐locus sequence typing (MLST) scheme, evolution can be considered neutral, or near neutral (Falush *et al*., 2003). Therefore, negative D values for these genes (Table 2), which would normally signify positive selection, are interpreted as signatures of population expansion events. All genes/domains within the *htrA* region showed negative values for Tajima's D, similar but slightly higher than those we have previously reported for MLST genes (Moodley *et al*., 2009; 2012).

**Table 2:** Genetic diversity and selection within the htrA gene region

Region/domain	Gene	*n*	Size^a^ (bp)	Polymorphism				Selection
				P	H	HD	π	D
HP1017 (*rocE*)	Amino acid permease	53	1560	291	50	0.998	0.04176	−0.50786
HP1018/1019 (*htrA*)	Serine protease	53	1428	296	50	0.997	0.04963	−0.10142
HP1020	Conserved hypothetical protein	53	1220	309	49	0.996	0.05928	−0.12746
HP1021	Response regulator	53	896	184	49	0.996	0.04343	−0.32504
HP1022	Predicted coding region	53	836	115	49	0.996	0.05209	−0.57713
HP1017‐1022	Combined region	53	6288	1296	51	0.999	0.05001	−0.31994
*atpA*		769	627	235	667	0.999	0.02974	−1.51646
*efp*		769	410	169	649	0.999	0.03389	−1.38453
*mutY*		769	420	214	677	0.999	0.06068	−0.88337
*ppa*		769	398	149	644	0.999	0.02500	−1.67487
*trpC*		769	456	273	686	0.999	0.07428	−0.83305
*ureI*		769	585	204	688	0.999	0.03005	−1.42932
*yphC*		769	510	254	701	0.999	0.04449	−1.35692

a In reference strain 26695.

P, number of polymorphic sites; H, number of haplotypes; HD, haplotype diversity; π, nucleotide diversity; Fs, Fu's Fs statistic; D. Tajima's D statistic.

**Figure 2:**
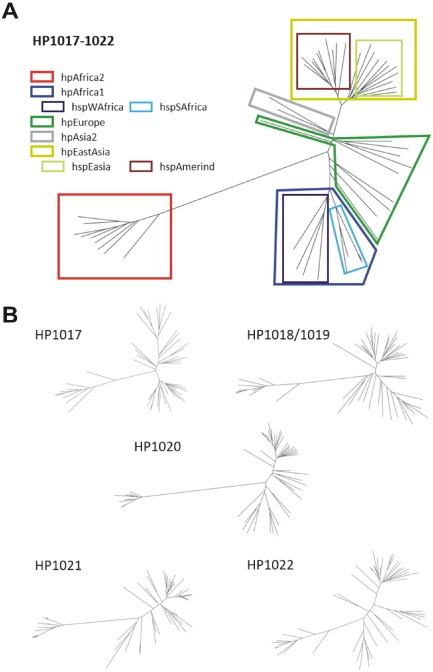
Global genetic structure and evolution of the htrA gene region. A. Unrooted neighbour‐joining phylogeny of the entire 6,288 bp htrA region showed that a worldwide sample of strains was structuring according to MLST populations (colour‐coded) and suggests evolution of the region mainly by genetic drift. Each gene/domain within the region (B) recovered highly similar topologies, implying that selection did not affect particular loci disproportionately.

Genetic variation across the *htrA* region was partitioned phylogenetically into the classical MLST‐defined populations (Fig. 2). Each gene/domain within the region also showed a very similar topology, which would be expected given their tight linkage. Taken together, these findings indicate that, similarly to the *H. pylori* MLST housekeeping genes, positive selection has had little to no effect on the *htrA* region over the course of *H. pylori*'s coevolution with humans. These results suggest that *htrA* is an important gene in *H. pylori*.

### Regulation of transcription in the htrA gene locus of *H* *. pylori*

**Figure 3:**
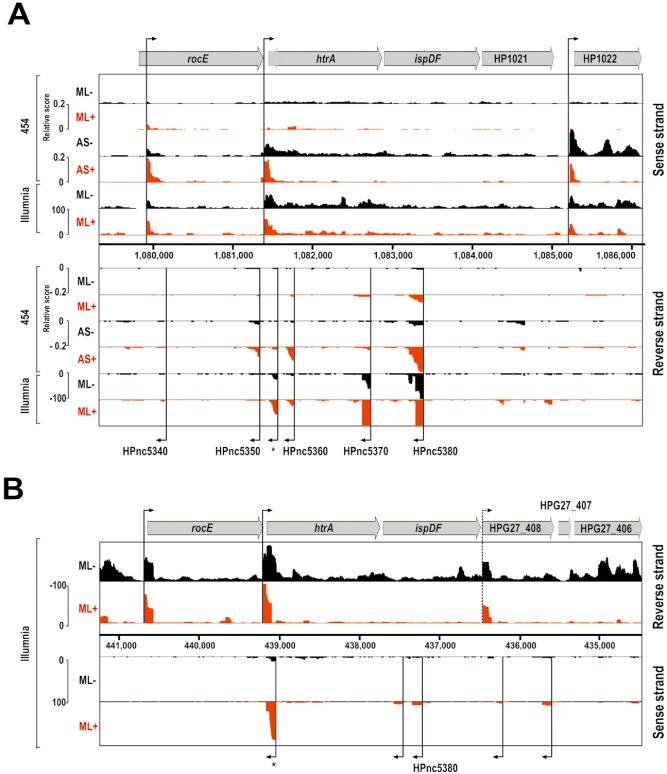
RNA‐seq based expression analysis of the htrA locus at exponential growth phase and under acidic stress conditions. A. Total RNA was isolated from *H* *. pylori* strain 26695 grown either to mid‐exponential growth phase (ML) or under acidic stress conditions (AS). DNase I‐treated RNA samples were used for the construction of cDNA library pairs: one denoted as (−), in which the total RNA was left untreated, and the other (+), in which total RNA was treated with terminator exonuclease treatment (TEX). High‐throughput sequencing of cDNA libraries was performed on either the Roche 454‐ or Illumina sequencing platform. The ML −/+ TEX and AS −/+ TEX 454 datasets were previously published (Sharma *et al*., 2010). New ML−/+ Illumina data were generated to increase sequencing coverage (26695: Bischler *et al*., 2015; G27: S. Pernitzsch and C. Sharma, unpublished). The generated cDNA sequencing reads without [black, (−) libraries] or with [red, (+) libraries] terminator exonuclease treatment were mapped to the htrA locus in the *H* *. pylori* 26695 chromosome and are visualised as coverage plots representing the number of reads (left scale, relative score) per nucleotide in the Integrated Genome Browser (IGB, Affymetrix). Grey arrows represent the annotated open reading frames and black arrows denote published transcriptional start sites (TSS) in *H* *. pylori* 26695 (Sharma *et al*., 2010). B. Mapped cDNA reads of + /− TEX‐treated cDNA libraries from *H* *. pylori* strain G27 mid‐log growth samples were aligned to the htrA locus. In contrast to 26695, a putative TSS (dotted arrow) was identified for HPG27_408 (HP1021), encoding an orphan response regulator. In both *H* *. pylori* strains, multiple *cis*‐encoded small RNAs (denoted HPnc) are encoded antisense to htrA locus. In addition to the previously published antisense RNAs (Sharma *et al*., 2010), a novel TSS was identified antisense to the 5′ coding region of htrA (denoted by a star).

The goal of the next set of experiments was to systematically investigate the transcription in the *htrA* gene locus (Sharma *et al*., 2010; Bischler *et al*., 2015). Differential RNA‐sequencing (dRNA‐seq) of two model *H. pylori* strains, 26695 and G27 (S. Pernitzsch and C. Sharma, unpublished), indicated that *htrA* is encoded as the first gene of an operon with two subsequent genes, *ispDF* and HP1021 (Fig. 3), encoding for a 2‐C‐methyl‐D‐erythritol 4‐phosphate cytidylyltransferase/2‐C‐methyl‐D‐erythritol 2,4‐cyclodiphosphate synthase and an orphan response regulator respectively (Donczew *et al*., 2015). Transcription of this polycistronic mRNA initiates from a transcriptional start site upstream of *htrA* and is induced under acidic stress conditions (Fig. 3, ML versus AS from 454‐sequencing data; Sharma *et al*., 2010). In contrast to *H. pylori* strain 26695, a putative transcriptional start site (TSS) was identified upstream of HPG27_408 (HP1021 homologue) in G27, suggesting transcriptional uncoupling of this response regulator from the *htrA*‐*ispDF* operon in this strain. Several potential *cis*‐encoded small RNAs, including HPnc5350, HPnc5360, HPnc5370 and HPnc5380, were found to be expressed antisense to *htrA* and *ispDF* in *H. pylori* strain 26695. At least three of them overlap with the coding region (HPnc5360, HPnc5370) or promoter region of *htrA* (HP5350). In addition, a previously uncharacterised TSS (TSS*, Fig. 3) was found to be expressed antisense to the putative leader of *htrA* mRNA. Similar to the *htrA*‐*ispDF*‐HP1021 operon, expression of these antisense RNAs is slightly induced when *H. pylori* 26695 was grown at low pH conditions (Fig. 3, compare ML versus AS from 454‐sequencing data). At least two of the potential small antisense transcripts (HPnc5380 and TSS*) were found to be conserved between *H. pylori* strains 26695 and G27. In addition, several strain‐specific antisense TSS were identified within the coding region of *htrA*, *ispDF* and HP1021 (data not shown).

### HtrA protein expression and secretion of active p165 and p220 oligomers

**Figure 4:**
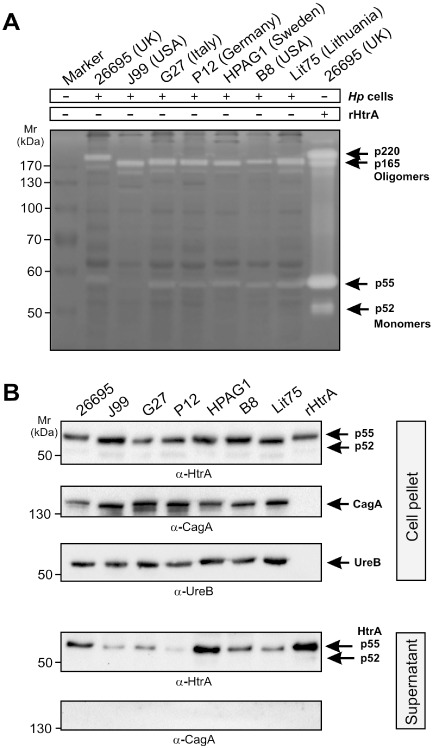
HtrA protein expression, oligomer formation and secretion in a large set of strains. A. Casein zymography of the indicated *H* *. pylori* strains reveals proteolytically active HtrA protein species of 55 kDa, 165 kDa and 220 kDa as labelled with arrows. Purified recombinant HtrA of 26695 is loaded in lane 9 and produced the same pattern of active HtrA forms. B. *H* *. pylori* isolates were grown in liquid BHI medium for 12 h and then fractionated in bacterial cell pellets (top) and supernatants (bottom). Immunoblotting using α‐HtrA antibodies show that all *H* *. pylori* strains exhibit strong HtrA signals in the supernatant fraction but varied substantially in their band intensities. As control, the bacterial pellets and supernatants were probed with α‐CagA and α‐UreB antibodies, excluding artificially lysed bacteria.

To analyse whether a large number of *H. pylori* strains produce proteolytically active HtrA protein species, we performed casein zymography of the corresponding bacterial lysates (Fig. 4A). In agreement with previous studies on *H. pylori* strain 26695 (Löwer *et al*., 2008), at least three casein‐cleaving proteases are produced by worldwide *H. pylori* strains, exhibiting apparent molecular weights of approximately 55 kDa (p55), 165 kDa (p165) and 220 kDa (p220) (Fig. 4A, arrows). The identity of these proteins as HtrA was approved by MALDI‐TOF mass spectrometry (Löwer *et al*., 2008). Interestingly, purified recombinant HtrA of 26695 produced the same pattern on casein gels, providing further evidence that these proteins represent HtrA forms (Fig. 4A, last lane). Because casein zymography was performed under non‐reducing conditions, the upper bands result from HtrA monomers forming oligomeric protein complexes. Also, protease DegP of *E. coli*, which is an orthologue of HtrA from *H. pylori*, was shown to form hexamers in their crystal structures (Krojer *et al*., 2002). In addition, in contrast to the double band detected for HtrA monomers by SDS‐PAGE and recombinant HtrA, we observed only a single proteolytic active p55 monomer in the majority of *H. pylori* lysates, whereas an active p52 was mostly not seen (Fig. 4A, lanes 2–8). To further test if the various *H. pylori* isolates secrete HtrA into the supernatant, all above described strains were grown for 12 h in brain heart infusion (BHI) liquid broth medium. Bacterial supernatants and cell pellets were prepared, and the presence of secreted HtrA proteins in the supernatant was investigated by immunoblotting using α‐HtrA antibodies (Fig. 4B). The results show that all *H. pylori* strains exhibit strong HtrA signals in the supernatant fraction but varied substantially in their band intensities (Fig. 4B, bottom). As control, the bacterial pellets and supernatants were probed with α‐CagA antibodies. The α‐CagA blots of bacterial cell pellets show strong bands of similar intensity as expected, confirming that equal amounts of protein were present in each sample (Fig. 4B, top), whereas the supernatants are devoid of CagA, indicating absence of lysed bacteria and cell debris (Fig. 4B, bottom). Taken together, these experiments confirm that the export of HtrA might occur via active signal peptide‐dependent translocation and following step(s), rather than being an artefact of bacterial autolysis in the *H. pylori* liquid culture.

### HtrA from multiple *H* *. pylori* strains cleave E‐cadherin

**Figure 5:**
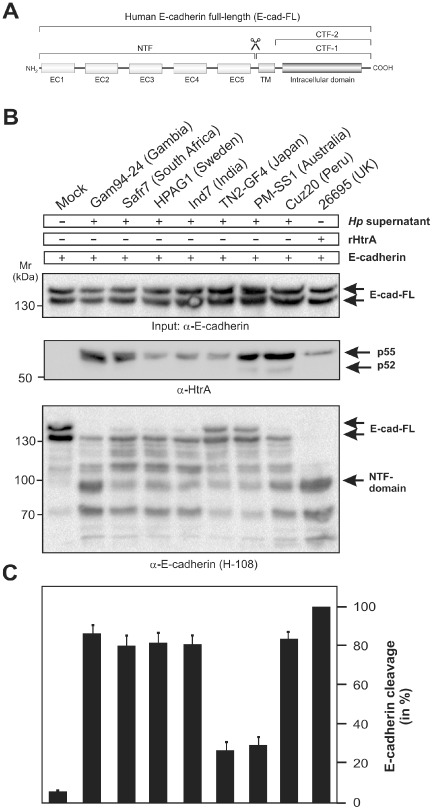
HtrA from multiple *H* *. pylori* strains cleave E‐cadherin. A. Domain structure of human E‐cadherin. Full‐length E‐cadherin (E‐cad‐FL) is a 130 kDa protein, which is composed of the 90 kDa extracellular domain amino‐terminal fragment (NTF) and 40 kDa carboxy‐terminal fragment (CTF‐1), containing the intracellular soluble 33 kDa CTF‐2 fragment. B. *In vitro* cleavage assays of purified E‐cadherin with the various HtrAs. HtrA from all tested strains cleaved E‐cadherin, as monitored by the degradation of E‐cad‐FL and appearance of the cleaved 90 kDa NTF fragment (arrows). C. Quantification of HtrA cleavage by the various HtrAs. Full cleavage of E‐cadherin was seen with recombinant HtrA of strain 26695 and was set 100% (last lane). Measurements were performed in triplicate.

We have recently shown that HtrA of strain 26695 can cleave the adherens junction protein E‐cadherin in polarised epithelial cells followed by paracellular transmigration of the bacteria (Hoy *et al*., 2010; 2012; Boehm *et al*., 2012). Full‐length E‐cadherin (E‐cad‐FL) is a 130 kDa protein, which can be cleaved in the 90 kDa extracellular domain amino‐terminal fragment (NTF) (Fig. 5A). Hence, we asked if HtrA from a large number of above described worldwide *H. pylori* strains can cleave E‐cadherin (Table S1 in the supporting information). To test this important question, we incubated secreted HtrA from *H. pylori* with recombinant human E‐cadherin. Addition of low concentrations of HtrA from all tested strains efficiently cleaved E‐cadherin, as monitored by the degradation of E‐cad‐FL and appearance of the cleaved 90 kDa NTF fragment (Fig. 5B, Table S1). However, the results also showed that the NTF band intensities varied substantially among the different strains, suggesting that strain‐specific differences exist in the activity of the various HtrAs on E‐cad‐FL (Fig. 5C). As a control, HtrA did not cleave recombinant EGFR or JAM (data not shown), indicating that E‐cadherin represents a selective substrate for *H. pylori* HtrA.

### Failure of htrA gene knockout in worldwide *H* *. pylori* strains

The generation of a defined *htrA* knockout mutant of *H. pylori* is particularly important to elucidate virulence mechanisms of this pathogen. In order to generate such mutant in the *htrA* gene, we applied a conventional protocol for the cloning of suicide double crossing‐over plasmids for typical *H. pylori* mutagenesis*.* The procedure and constructs are outlined in Fig. S3 in the supporting information. First, we cloned a subset of *htrA* insertion constructs using chloramphenicol (Fig. S3A) or kanamycin resistance gene cassettes (Fig. S3B). Second, we cloned a subset of *htrA* deletion constructs using the kanamycin cassette replacing either the full‐length *htrA* gene (Fig. S3C) or the *htrA* signal peptide sequence (Fig. S3D). These plasmids were transferred into a large set of recipient strains (21 isolates listed in Table S1 in the supporting information and 75 strains described by Backert *et al*., 2004) using the natural transformation (Fernandez‐Gonzalez and Backert, 2014) or electroporation (Segal and Tompkins, 1993) techniques. The corresponding antibiotics‐containing GC agar plates were incubated at 30°C, 34°C or 37°C and were checked for potential mutant colonies after one, two and three weeks. Using this common strategy, we did not get any chloramphenicol‐ or kanamycin‐resistant *htrA* mutant clones. In rare cases, we sporadically obtained a very few colonies after about 1 week, but PCR of the resulting DNA showed that the antibiotics resistance cassettes were not incorporated into the *htrA* gene but randomly elsewhere in the chromosome (data not shown). These results suggest that *htrA* cannot be mutagenised in *H. pylori* by conventional methods.

### Mutagenesis and complementation of genes flanking htrA

**Figure 6:**
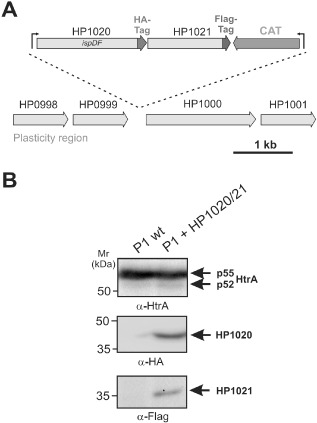
Genetic complementation and expression of HP1020 and HP1021 genes in the operon downstream of htrA. A. Schematic presentation of the complementation construct used to express the HP1020 and HP1021 genes (top) in the plasticity region of *H* *. pylori* (bottom). The expression construct at the top is composed of the htrA gene promotor followed by the HP1020 and HP1021 genes that are fused to HA‐ and FLAG‐tags, as indicated. At the 3′ end, a chloramphenicol resistance gene cassette was added to select clones. B. This construct was transformed into *H* *. pylori* and chloramphenicol‐resistant colonies were screened for HA‐ and FLAG‐tagged proteins with the expected sizes of HP1020 and HP1021 proteins respectively. The corresponding wild‐type *H* *. pylori* strains did not reveal signals with these antibodies as expected. The α‐HtrA blot served as control, showing that equal amounts of protein are loaded in each lane.

The existence of any polar effects in some of our knockout constructs cannot be ruled out completely. Thus, we next aimed to investigate if we can mutate all other genes in the *htrA* operon or if one of them is maybe lethal. For this purpose, the corresponding genes of HP1020 and HP1021 were cloned, and knockout constructs were generated by insertion of the kanamycin‐resistance gene cassette (Fig. S4 in the supporting information). Interestingly, we were able to obtain mutant clones from each of the two genes by natural transformation at frequencies between 1.4 and 3.5 × 10^−7^ (Fig. S4A–B). This confirms that our mutagenesis protocols work efficiently and excludes the possibility that polar effects on any of those genes are responsible for the failure to mutate *htrA*. To further corroborate these findings, we expressed second copies of the HP1020 and HP1021 genes of the *htrA* operon under the direct control of the *htrA* promotor and tagged the genes with haemagglutinin (HA)‐ and FLAG‐sequences to monitor the proper expression of corresponding proteins. This fusion construct was introduced in the plasticity region of *H. pylori* (between ORFs HP0999 and HP1000) using a strategy shown in Fig. 6A. The correct expression of the corresponding genes was verified by Western blotting (Fig. 6B). *H. pylori* clones from these complementants were then subjected to natural transformation and electroporation with all *htrA* kanamycin‐based knockout constructs shown in Fig. S3. Again, we were unable to obtain *htrA* mutant clones, supporting the hypothesis that *htrA* may be an essential gene in *H. pylori*.

### Evidence for htrA representing an essential gene in *H* *. pylori*

**Figure 7:**
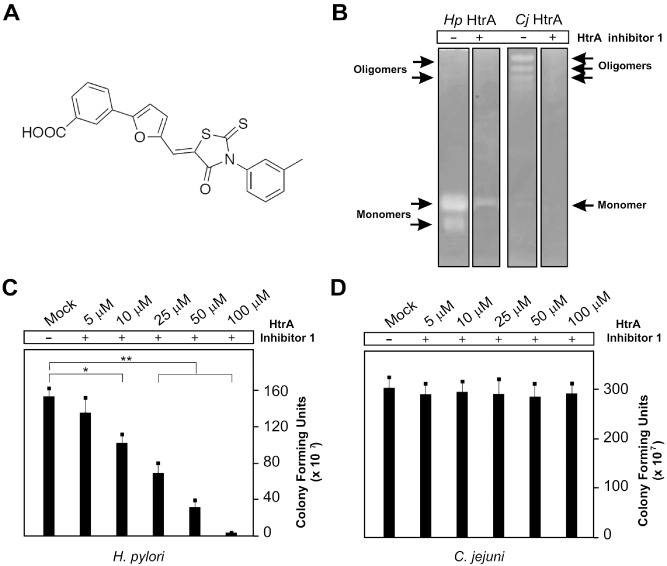
Evidence for htrA representing an essential gene in *H* *. pylori*. A. Structure of the recently developed HtrA inhibitor (Perna *et al*., 2015). B. We used this HtrA inhibitor to study its effect on bacterial survival. Control experiments confirm that 50 μM inhibitor compound‐1 inhibits HtrA activity of *H* *. pylori* and *C* *. jejuni in vitro* using casein zymography of bacterial pellets. *H* *. pylori* and *C* *. jejuni* were grown in BHI medium in the presence or absence of different concentrations of the inhibitor as indicated and centrifuged. In the mock control, we added DMSO. After 12 h incubation, the cfu was determined for *H* *. pylori* on GC agar plates (C) and for *C* *. jejuni* on MH agar plates (D). The results show that inhibition of HtrA enzyme activity significantly inhibits the growth of *H* *. pylori* but not *C* *. jejuni*. The experiments were done in triplicate.

To obtain final proof that *htrA* is an indispensible gene and to investigate if its serine protease activity is necessary for *H. pylori*, we next focused on experiments using pharmacological inhibition of HtrA. We have recently performed a structure‐based virtual screening for small‐molecule inhibitors of HtrA in *H. pylori* and *C. jejuni*. After screening of compound databases and biochemical tests, we identified HtrA inhibitor compound‐1 as a novel small‐molecule inhibitor that efficiently blocked E‐cadherin cleavage *in vitro* (Perna *et al*., 2015) (Fig. 7A). When *H. pylori* HtrA was incubated with recombinant E‐cadherin and increasing amounts of the inhibitor, 50 μM concentration was sufficient to reduce HtrA‐mediated E‐cadherin cleavage significantly (Perna *et al*., 2015). In agreement with these findings, we observed inhibition of proteolytic activity of HtrA from *H. pylori* and *C. jejuni* as visualised by casein zymography (Fig. 7B). Some remaining minor bands correspond to HtrA‐independent other proteases (Löwer *et al*., 2008). Interestingly, we found that HtrA inhibitor compound‐1 can kill *H. pylori* in a dose‐dependent manner (Fig. 7C), while it had no effect on the growth of *C. jejuni* in parallel experiments (Fig. 7D). We can also show that compound‐1 did not affect the growth of *Salmonella typhimurium* and *Shigella flexneri* (Fig. S5 in the supporting information). These observations are in line with the conclusion that *htrA* is an essential gene in *H. pylori* requiring its protease activity, whereas *htrA* is not essential in *C. jejuni*, *S. typhimurium* or *S. flexneri* and can be inactivated in various strains by mutagenesis (Humphreys *et al*., 1999; Purdy *et al*., 2002; Mo *et al*., 2006; Boehm *et al*., 2012; Hoy *et al*., 2012).

## Discussion

Knowledge is accumulating that bacteria secrete proteases with multiple roles in microbial pathogenesis, but information on secreted *H. pylori* proteases and their function is still restricted. In many bacteria, HtrA is a well‐known periplasmic protein that exhibits both chaperone and proteolytic activities with important roles in protein quality control critical for stress tolerance and bacterial survival (Clausen *et al*., 2002; Ingmer and Brøndsted, 2009; Backert *et al*., 2013; Frees *et al*., 2013; Skorko‐Glonek *et al*., 2013). Moreover, it has been shown that HtrA is critical for the pathogenicity of various bacterial pathogens including *Campylobacter*, *Chlamydia*, *Klebsiella, Listeria*, *Salmonella*, *Shigella* and *Yersinia* species (Li *et al*., 1996; Humphreys *et al*., 1999; Cortés *et al*., 2002; Purdy *et al*., 2002; Wilson *et al*., 2006; Gloeckl *et al*., 2013; Heimesaat *et al*., 2014a, 2014b). However, *htrA* does not seem to be an essential gene in these bacteria because the generation of Δ*htrA* deletion mutants has been reported. By contrast, generation of a mutant of the *htrA* gene in *H. pylori* has failed in a few strains but the circumstances remained unknown (Salama *et al*., 2004; Hoy *et al*., 2010). Here we show that the genetic organisation of *htrA* and flanking genes, *htrA* transcription and function is conserved in worldwide *H. pylori* isolates, and provide direct evidence that inactivation of the gene and inhibition of its protease activity is lethal for these bacteria.

We found that the *htrA* gene is present and conserved in worldwide *H. pylori* strains. For this purpose, we collected a very large group of *H. pylori* DNAs from gastric biopsies of 992 patients across the planet, from Germany, Japan, Singapore, USA, Mexico and Colombia respectively. Using PCR, we verified that the *htrA* gene is present in all isolates. Furthermore, we investigated in detail the entire *htrA* gene locus in 55 fully sequenced *H. pylori* strains and observed a very similar gene topology, which would be expected given their tight linkage. The genetic organisation and gene order, with six genes/domains (HP1017 to HP1022), appears to be highly conserved. Conversely, the *htrA* genes among these strains were highly variable at the nucleotide level, containing altogether 1,296 polymorphic sites. Diversity within each gene/domain was very similar for haplotype and nucleotide diversity, respectively, and the genetic variation across the *htrA* locus was partitioned phylogenetically into the classical MLST‐defined *H. pylori* populations in the world (Linz *et al*., 2007; Moodley *et al*., 2009; 2012). Each gene/domain within the region also showed similarity to the *H. pylori* MLST housekeeping genes, suggesting that positive selection had only minor effects on the *htrA* region during the long coevolution of *H. pylori* with its human host. That the entire *htrA* region is structured very similarly to the MLST genes, being present in all six studied global populations, also implies that *H. pylori*'s acquisition of the region most likely predates its association with humans. These analyses provided first direct evidence towards *htrA* being an important gene in *H. pylori*.

We found that not only the genetic organisation of the *htrA* gene locus is highly conserved in *H. pylori*, but also a series of other important features. First, RNA‐sequencing of two *H. pylori* strains showed that *htrA* is encoded as the first gene of an operon with two subsequent genes, *ispDF* and HP1021. This operon structure is also conserved in all other fully sequenced *H. pylori* strains. Transcription of the resulting polycistronic mRNA initiates from a conserved transcriptional start site upstream of the *htrA* gene and is enhanced under high acid conditions, which is in agreement with the role of *htrA* in mediating stress resistance. Second, once the HtrA protein is produced by the bacteria, it is delivered into the periplasm and secreted into the extracellular space. This important new feature seems also conserved among a wide range of *H. pylori* strains worldwide (Table S1 in the supporting information). HtrAs in Gram‐negative bacteria such as *H. pylori* contain a signal peptide important for Sec‐dependent cleavage and transport of the protease across the inner membrane into the periplasm (Clausen *et al*., 2002; Ingmer and Brøndsted, 2009; Frees *et al*., 2013; Skorko‐Glonek *et al*., 2013), while its transport across the outer membrane remains unclear. Commonly, HtrAs exhibit no sequence homology to typical autotransporters and process themselves by autoproteolysis. This is in line with our observation that the HtrA protease activity in a closely related bacterium, *C. jejuni*, is not required for its secretion (Boehm *et al*., 2013). Thus, HtrA very likely requires the assistance of other bacterial factors for delivery. Candidates are secretion systems called type I‐VII (T1SS‐T7SS), but various available *H. pylori* genome sequences do not encode typical orthologues, except the well‐known T4SS in the *cag*PAI, which is dispensable for HtrA secretion (Löwer *et al*., 2008). Forthcoming experiments should therefore study in detail how HtrA is secreted by *H. pylori*. Third, we tested if the *H. pylori* strains can generate proteolytic active HtrA oligomers. Using casein zymography, we could demonstrate that all tested *H. pylori* strains formed caseinolytic active oligomers with expected sizes of trimers and higher. These observations are in agreement with reports on HtrA in other bacteria such as *E. coli*, where the HtrA oligomers are highly proteolytic active rather than the monomer (Krojer *et al*., 2008). These oligomers were found both in total cell lysates and culture supernatants of *H. pylori.* Fourth, we could demonstrate that secreted HtrA from a large collection of worldwide strains can cleave the tumour‐suppressor and junctional protein E‐cadherin. HtrA may therefore interfere with epithelial barrier function and gastric disease development as the protease effectively destroys adherens junctions in polarised epithelial cells *in vitro* and during infection (Hoy *et al*., 2010; 2012; Boehm *et al*., 2012).

Finally, we provided several lines of evidence that *htrA* is an essential gene in *H. pylori*. Our genetic mutagenesis and complementation studies of each of the genes in the *htrA* operon revealed that *ispDF* and HP1021, but not *htrA*, can be mutated, thus excluding polar effects. We also considered that potential *H. pylori htrA* mutants maybe temperature‐sensitive or grow very slowly compared with wild‐type bacteria. However, incubation of transformed *H. pylori* cultures at 30°C, 34°C or 37°C for up to 3 weeks did not reveal any mutant clones. Although we tested 96 worldwide strains and were unable to mutagenize *htrA*, we cannot exclude the possibility that mutagenesis maybe possible in some others. We must therefore screen more strains in future. In addition, we need to identify the intracellular HtrA targets to understand its essentiality in the majority of strains. Finally, novel strategies must be developed to block HtrA functions in *H. pylori*. One such option is the development of HtrA‐specific inhibitors. For this purpose, we developed a small inhibitor compound and demonstrate that suppression of HtrA proteolytic activity is sufficient to effectively kill *H. pylori*, but not other bacteria such as *C. jejuni*, *S. typhimurium* and *S. flexneri*.

Taken together, HtrA is a well‐known periplasmic protein exhibiting chaperone and proteolytic activities controlling stress responses, bacterial survival and virulence properties. However, according to current opinion, *htrA* is no essential gene in the bacterial world (Johnson *et al*., 1991; Elzer *et al*., 1994; Li *et al*., 1996; Humphreys *et al*., 1999; Cortés *et al*., 2002; Purdy *et al*., 2002; Brøndsted *et al*., 2005; Wilson *et al*., 2006). Surprisingly, we show here that *Helicobacter* HtrA is an essential bifunctional protein with crucial intracellular and extracellular functions. We demonstrate that the genetic organisation of *htrA* and flanking genes, *htrA* transcription and extracellular function for E‐cadherin cleavage is preserved in *Helicobacter* isolates around the globe and provide direct evidence that *htrA* inactivation is lethal for these bacteria. Thus, we describe here the first microbe in which *htrA* is an indispensable gene, a situation unique in the bacterial kingdom. HtrA can therefore be considered a promising new target for anti‐bacterial therapy. It is evident that future research should be devoted to designing and optimising a more potent, selective and specific inhibitor of *H. pylori* HtrA. Inhibitor compound‐1 represents a first point of reference for virtual docking studies, which should aim at selectivity with regard to other serine proteases and an improved potency. Ideally, new ‘drug‐like’ scaffold structures will be retrieved in future experiments, such as those now being performed in our laboratory.

## Experimental procedures

### Patients and isolation of DNA from biopsies

Genomic DNA from 992 *H. pylori*‐positive gastric biopsies was isolated (Table 1).

#### German samples

In this study, 187 ambulatory patients with abdominal complaints underwent routine gastrointestinal endoscopy (Rad *et al*., 2002). Their mean age was 64.2 years, ranging from 23 to 92 years; 88% were originally from Germany and 12% were living in Germany but immigrated from other European countries. Patients taking non‐steroidal anti‐inflammatory drugs or receiving anti‐secretory therapy were excluded from this study. Five antral biopsies were collected from each of 187 consecutively enrolled patients after informed consent. Two antral sections were evaluated for histopathological parameters, and the three remaining biopsy specimens were stored in liquid nitrogen, homogenised, and DNA was isolated using the QIAamp DNA Mini Kit (Qiagen) according to the manufacturer's protocol. The corresponding ethics votum was approved by the Medical Faculty Ethics Commission of Technical University Munich (project number 2453/09).

#### Singapore samples

The 80 *H. pylori* strains were isolated from gastric biopsies obtained from the gastric antrum within 2 cm of the pylorus in patients who underwent upper gastrointestinal endoscopy at the National University Hospital, Singapore. Informed consent was obtained from all patients for gastroscopy and biopsies. Patients were enrolled from outpatients attending the gastroenterology clinic who were scheduled for upper gastrointestinal endoscopy. Exclusion criteria were age younger than 21 years or older than 80 years, previous upper gastrointestinal surgery, or ingestion of antibiotics, proton pump inhibitors or non‐steroidal anti‐inflammatory drugs within 4 weeks of endoscopy. The *H. pylori* strains were cultured on chocolate blood agar plates supplemented with 5% lysed horse blood at 37°C in an atmosphere of 5% CO_2_ in a humidified incubator. Chromosomal DNA was extracted from 3‐day‐old cultures using the QIAamp DNA Mini Kit (Qiagen) according to the manufacturer's protocol.

#### Mexican samples

Two hundred three samples were from patients attending the Gastroenterology Unit of the Mexico General Hospital (Secretarıa de Salud) and the Oncology Hospital (Instituto Mexicano del Seguro Social), which are both hospitals in Mexico City. Patients were older than 30 years, consulted because of gastroduodenal symptoms (General Hospital) or because of a probable gastric cancer (Oncology Hospital), and were programmed for endoscopy and biopsy for diagnostic purposes. Subjects who had previously received cancer treatment were on antibiotics, anti‐*H. pylori* therapy or non‐steroidal anti‐inflammatory drugs 2 weeks prior to the study or had other severe chronic diseases were excluded. DNA was extracted from frozen tissues using QIAamp DNA Micro Kit (Qiagen, Hilden, Germany) according to the manufacturer's instructions.

#### US, Japanese and Colombian samples

The 201 *H. pylori* strains from the US, 152 from Japanese and 169 from Colombian population were isolated from gastric biopsies obtained from the gastric antrum within 2 cm of the pylorus in patients who underwent upper gastrointestinal endoscopy at the Michael E. DeBakey Veterans Affairs Medical Center, Houston, Texas, US, Kyoto Prefectural University of Medicine Hospital, Kyoto, Japan, and Universidad Nacional de Colombia, Bogota, Colombia respectively. Informed consent was obtained from all patients for gastroscopy and biopsies. Patients were enrolled from outpatients attending the gastroenterology clinic who were scheduled for upper gastrointestinal endoscopy. Exclusion criteria were patients with previous upper gastrointestinal surgery, or ingestion of antibiotics, proton pump inhibitors or non‐steroidal anti‐inflammatory drugs within 4 weeks of endoscopy. *H. pylori* strains were grown at 37°C on BHI (Difco) plates containing 7% horse blood (Cocalico Biological, Reamstown, PA) in a 12% CO_2_ incubator with 100% relative humidity. The organisms were identified as *H. pylori* by Gram staining, colony morphology, and positive oxidase, catalase and urease reactions. Multiple isolates on the plates were pooled together, and genomic DNA was extracted with the QIAamp tissue kit (Qiagen, Santa Clarita/CA) according to the manufacturer's instructions. All strains were selected randomly from culture stocks that were used previously for genotyping studies (Yamaoka *et al*., 1998; 1999; Lu *et al*., 2005).

### Bacterial strains and growth conditions

The *H. pylori* strains employed in this study include 21 fully sequenced isolates (Table S1 in the supporting information) and 75 German strains (Backert *et al*., 2004). *H. pylori* were grown on GC agar (Oxoid) plates supplemented with 10% donor horse serum (Biochrom AG), vitamin mix (1%), vancomycin (10 μg ml^−1^), nystatin (1 μg ml^−1^) and trimethoprim (5 μg ml^−1^) (Kumar Pachathundikandi *et al*., 2011; Wiedemann *et al*., 2012). All antibiotics were obtained from Sigma‐Aldrich. Incubation of the bacteria was performed at 37°C for 2 days in anaerobic jars containing Campygen^TM^ gas mix (Oxoid, Germany) (Patel *et al*., 2013; Tenguria *et al*., 2014). *C. jejuni* strain 81–176, *S. typhimurium* SL1344 and *S. flexneri* SA100 were described (Humphreys *et al*., 1999; Purdy *et al*., 2002; Boehm *et al*., 2011; Krause‐Gruszczynska *et al*., 2011). The bacterial concentration in BHI medium (Becton, Dickinson and Company) was measured as optical density (OD) at 550 nm using an Eppendorf spectrophotometer (Kim *et al*., 2013). Serial dilutions of the bacterial cultures were made, and the colony‐forming units (cfu) were quantified in the presence or absence of inhibitor compound‐1 (Perna *et al*., 2015).

### HtrA secretion assays

*Helicobacter pylori* strains were suspended in BHI medium supplemented with 10% FCS. The optical density was determined and adjusted to OD_550_ = 0.2. To allow HtrA secretion in the culture supernatant, the bacteria were incubated for 12 h shaking at 160 r.p.m. The cell pellets and the supernatants were separated by centrifugation at 4,000 r.p.m. The supernatants were then passed through 0.21 μm sterile filters (Sigma Aldrich, Taufkirchen/Germany) to remove remnant bacterial cells. Absence of live bacteria in the supernatant was verified by the lack of CFU after 3 days of incubation on GC agar plates. The obtained bacterial pellets and supernatants were then analysed by Western blotting and casein zymography as described below.

### Evolutionary analysis

A whole genome sequence alignment of 53 *H. pylori* strains, accounting for six of the seven populations known globally from MLST analyses, was created in Mauve (Darling *et al*., 2004), from which the entire locus (6,288 bp) was extracted. Genetic diversity and Tajima's population parameter D, which can detect departures of gene sequences from neutrality (Tajima, 1989), were calculated for the entire HtrA region, for each defined gene/domain within the region and for each MLST gene from the worldwide sample of 769 isolates originally published by Linz *et al*. (2007) using DNAsp (Rozas *et al*., 2003).

Furthermore, the HtrA region was analysed phylogenetically to determine whether genetic drift had caused variation within the region to be subdivided on the basis of geography, as reported for essential MLST housekeeping genes (Linz *et al*., 2007), or whether selection caused the locus to deviate from neutrality. Each gene/domain within the locus was also analysed separately to investigate if variation at certain genes was structured differently to the general locus topology. Therefore, the phylogenetic model parameters were determined in MEGA6 (Tamura *et al*., 2013), and each alignment was subjected to unrooted phylogenetic analysis using neighbour joining and the maximum‐likelihood determined model parameters in SplitsTree (Huson, 1998). All gene/domain subset alignments were analysed similarly.

### Helicobacter preparation for transcriptome analyses

*Helicobacter pylori* strains 26695 and G27 were initially grown as described above. Bacteria were re‐streaked every day to fresh plates to avoid coccoid cell formation. For liquid cultures, 15 or 50 ml BHI medium supplemented with 10% FBS (Biochrom AG) and above antibiotics were inoculated with *Helicobacter* from plate to a final OD_600 nm_ of 0.02–0.05 and grown under agitation at 140 r.p.m. in 25 cm^2^ or 75 cm^2^ cell culture flasks (PAA). Bacteria were grown at 37°C in a HERAcell 150i incubator (Thermo scientific) in microaerophilic environment (10% CO_2_, 5% O_2_ and 85% N_2_). Detailed information about the bacterial growth conditions (mid‐log growth and acidic stress) that have been previously used for the analysis of the primary transcriptome of *H. pylori* strain 26695 was described (Sharma *et al*., 2010).

### RNA extraction, cDNA library construction and illumina sequencing

For whole transcriptome analysis based on dRNA‐Seq, total RNA from *H. pylori* strains 26695 and G27 grown in liquid culture to mid‐exponential growth phase (OD_600nm_ ∼ 0.7 to 1.0) was extracted using the hot phenol method (Blomberg *et al*., 1990) and treated with DNase‐I (Fermentas) to remove genomic DNA. The dRNA‐seq approach discriminates primary form processed transcripts by sequencing different cDNA library pairs: one library denoted (−) from untreated total bacterial RNA, and the other (+) enriched for primary transcripts by terminator exonuclease treatment that specifically degrades transcripts with 5′ monophosphate but not those containing 5′ triphosphate (for details, see Sharma *et al*., 2010; Bischler *et al*., 2015). Therefore, RNA was either left untreated or subjected to terminator‐5′‐phosphate‐dependent exonuclease (TEX, Epicentre Biotechnologies) treatment prior to cDNA library construction. In general, cDNA libraries were generated by *vertis* Biotechnology AG, Germany, as described by Sharma *et al*. (2010). Following quality control, cDNA libraries were sequenced using either an Illumina Genome Analyser IIx or HiSeq2000 sequencer. Computational analysis of raw sequencing data was performed by the automated RNA‐seq processing pipeline READemption (Förstner *et al*., 2014). For data visualisation, graphs representing the number of mapped reads per nucleotide were calculated and visualised using the Integrated Genome Browser (IGB) version 6.5.3 software from Affymetrix (http://genoviz.sourceforge.net/). The whole transcriptome data set for strain G27 using Illumina sequencing as well as further details of read mapping and sequencing statistics will be published elsewhere (S. Pernitzsch and C. Sharma, University of Würzburg).

### Cloning and purification of the HtrA protein

Cloning and purification of HtrA from *H. pylori* strain 26695 was done as described (Löwer *et al*., 2008). Briefly, the *htrA* gene of ORFs HP1018/1019 was amplified from genomic DNA. The amplified *Bam*HI/*Eco*RI flanked PCR product was then ligated into the pGEX‐6P‐1 plasmid (GE Healthcare Life Sciences) and transformed in *E. coli* strain BL21. For purification of GST‐HtrA, transformed *E. coli* was grown in 500 ml TB medium to an OD_550nm_ of 0.6, and the expression was induced by the addition of 0.1 mM isopropylthiogalactosid (IPTG). The bacterial culture was pelleted at 4,000× *g* for 30 min and lysed in 25 ml PBS buffer by sonification. The lysate was cleared by centrifugation, and the supernatant was incubated with glutathione sepharose (GE Healthcare Life Sciences) at 4°C over night. HtrA was eluted with 180 U Prescission Protease for 16 h at 4°C (GE Healthcare Life Sciences). Cleavage products were analysed by SDS‐PAGE and zymography as described (Löwer *et al*., 2008; Hoy *et al*., 2010).

### Casein zymography

Undiluted aliquots were loaded onto 8% SDS‐PAGE gels containing 0.1% casein (Invitrogen, Germany) and separated by electrophoresis under denaturing conditions. After protein separation, the gel was re‐natured in 2.5% Triton X‐100 solution at room temperature for 60 min with gentle agitation, equilibrated in developing buffer (50 mM Tris‐HCl, pH 7.4; 200 mM NaCl, 5 mM CaCl_2_, 0.02% Brij35) at room temperature for 30 min with gentle agitation and incubated overnight at 37°C in fresh developing buffer. Transparent HtrA bands having caseinolytic activity were visualised by staining with 0.5% Coomassie Blue R250 as described (Löwer *et al*., 2008).

### *In vitro* cleavage assays of E‐cadherin

For *in vitro* cleavage studies, 50 ng recombinant human E‐cadherin (R&D Systems) was incubated with 50 ng purified HtrA. Alternatively, *H. pylori* strains were grown in BHI medium, and supernatants were prepared as described above. Five microlitres of cell‐free supernatants was mixed with 25 μl HEPES buffer (50 mM, pH 7.4) containing 50 ng E‐cadherin. All cleavage reactions were carried out at 37°C for 16 h.

### HtrA primer design

To verify *htrA* gene presence in *H. pylori* of different geographic origin, six primers (A–F) were designed to amplify various regions of the *htrA* gene as shown in Table S1 in the supporting information. Primer pair A and F (A: 5′‐ATG AAA AAA ACC CTT TTT ATC TCT‐3′; F: 5′‐TCA TTT CAC CAA AAT GAT CCT AT‐3′) amplify the complete *htrA* gene resulting in a 1,428 bp PCR product. Primers A and B (A: 5′‐ATG AAA AAA ACC CTT TTT ATC TCT‐3′; B: 5′‐GGG GTC ATT AAA CAC ACC G‐3′) generate a 231 bp product near to the N‐terminus of the *htr*A gene. To amplify a 573 bp fragment from the protease domain the primer set C and D was used (C: 5′‐TTT TTC CAA CAA TTT TTT GGG G‐3′; D: 5′‐GGT TTT GAT GAG TTG GGT TAC‐3′) and the primers E and F (E: 5′‐GGT AAG ATT GAA AGA GGT TAC‐3′; F: 5′‐TCA TTT CAC CAA AAT GAT CCT AT‐3′) amplify 624 bp of the C‐terminus including both PDZ domains of HtrA. All primers were purchased from Eurofins MWG GmbH (Ebersberg, Germany).

### PCR conditions

The different regions of the *htrA* gene were amplified using the primers A–F. The PCR reaction mixture (50 μl) contained 50 ng of genomic DNA, 1 U *Taq* polymerase (Qiagen, Hilden), 200 μM of each dNTP, 0.2 μM of each primer and 1× *Taq* polymerase buffer (supplied by the manufacturer). The following conditions were used for amplification: for primer pairs A–F/C–F, an initial denaturation for 5 min at 94°C, followed by 30 cycles of 1 min at 94°C, 30 s at 60°C and 2 min at 72° was performed. A final extension was done for 10 min at 72°C. For the primer sets A–D/C–D/E–F the elongation time was reduced to 1 min at 72°C and for the primers A–B the annealing step was adjusted to 30 s at 58°C and the elongation time to 30 s at 72°C. PCR products were separated on a 1.5% (w/v) agarose gel.

### Cloning of mutagenesis constructs

For the replacement of *htrA*, the 5′flanking region of *hp1017* was amplified using following primer: 5′‐AAA GAC CTC GGG TTT GGT TCT ATT TTT GA‐3′ and 5′‐AAA TCT AGA ATT TGC TAA ACT TCA AAA TT‐3′ with *Sac*I and *Xba*I restriction sites and the 3′flanking regions of *hp1020* using following primer: 5′‐AAA GTC GAC GTT AGT CGC ATG TCT TTG AT‐3′ and 5′‐AAA GGG CCC CGC TCC TAA AAT CGC ATC AA‐3′ with *Acc*I and *Apa*I restriction sites. The PCR products were inserted into pBluescript II KS (+) vector. A terminator‐less kanamycin resistance cassette and its promoter has been inserted via *Xba*I and *Pst*I sites. For the deletion of the *htr*A signal peptide (amino acid 1 to amino acid 17), the kanamycin resistance cassette was inserted into the pBluescript II KS (+) vector flanked by sequences of *hp1017* (as described above) and *hp1019*, which was amplified using the primer: 5′‐GTC GAC ATG GGC AAT ATC CAA ATC CAG AG‐3′ and 5′‐GGG CCC CTA TCA TTT CAC CAA AAT GA‐3′. We also produced two conventional insertion mutants. In these cases, the *htrA* gene was opened using the singular AgeI restriction site followed by insertion of the chloramphenicol or kanamycin resistance cassette respectively. All four *htrA* constructs are shown schematically in Fig. S3 of the supporting information.

For genetic complementation of HP1020 and HP1021 genes, we chemically synthesised a 2,409 bp DNA fragment comprising both genes under the control of a 130 bp *htrA* promotor sequence (Invitrogen). To monitor proper expression of corresponding proteins, we fused the HP1020 gene at the 3′ end with haemagglutinin (HA)‐tag (5′‐TAT CCG TAT GAT GTT CCT GAT TAT GCT‐3′) and HP1021 with FLAG‐tag (5′‐GAT TACAAG GAT GAC GAC GAT AAG‐3′) sequences. This fusion construct was introduced in the plasticity region of *H. pylori* strain P1 (between ORFs HP0999 and HP1000) using a strategy shown in Fig. 6A. At the 3′ end, a chloramphenicol resistance gene cassette was added to select clones. The correct expression of the corresponding HP1020/21 genes was verified by Western blotting as described below. We also produced two conventional insertion mutants for HP1020 and HP1021, respectively, using the kanamycin resistance cassette as shown in Fig. S4 of the supporting information.

### SDS‐PAGE and immunoblotting analysis

Bacterial pellets or cleavage reactions were mixed with equal amounts of 2× SDS‐PAGE buffer and boiled for 5 min. Proteins were separated by SDS‐PAGE on 8% polyacrylamide gels and blotted onto PVDF membranes (Immobilon‐P, Millipore, USA) as described (Lind *et al*., 2014). Before addition of the antibodies, membranes were blocked in TBS‐T (140 mM NaCl, 25 mM Tris‐HCl pH 7.4, 0.1% Tween‐20) with 3% BSA or 5% skim milk for 1 h at room temperature (Zhang *et al*., 2014). HtrA proteins were detected by a rabbit polyclonal α‐HtrA antiserum that was raised against the N‐terminal peptide (C‐DKIKVTIPGSNKEY) of HtrA, and Urease B subunit proteins were detected by a rabbit polyclonal α‐UreB antiserum that was raised against the N‐terminal peptide (C‐HDYTIYGEELK) of urease (Biogenes GmbH) (Tegtmeyer *et al*., 2013). CagA proteins were detected by incubation of the membranes with a rabbit polyclonal α‐CagA antibody (Austral Biologicals, USA). Monoclonal antibodies HecD1 and H‐108 against the extracellular domain of E‐cadherin were obtained from Calbiochem and Santa Cruz respectively. Anti‐HA (YPYDVPDYA) and anti‐FLAG (DYKDDDDK) antibodies were purchased from Cell Signaling Technology and Sigma‐Aldrich respectively. As secondary antibodies, horseradish peroxidase‐conjugated α‐mouse or α‐rabbit polyvalent rabbit and pig immunoglobulin, respectively, were used (Life Technologies, Germany). Antibody detection was performed with the ECL Plus chemiluminescence Western Blot system for immunostaining (GE Healthcare Life Sciences, Germany) (Hirsch *et al*., 2012; Roure *et al*., 2012).

### Statistics

All data were evaluated via Student's *t*‐test with SigmaPlot statistical software (version 13.0). Statistical significance was defined by *P* = 0.01 (*) and *P* = 0.001 (**).

## Supporting information

Supporting information
