# Supplementary material for: Characterisation of worldwide Helicobacter pylori strains reveals genetic conservation and essentiality of serine protease HtrA
Source: Mol Microbiol. 2015 Dec 22;99(5):925–44. doi: 10.1111/mmi.13276 (PMC4832355; doi:10.1111/mmi.13276)

Supplemental Table S1: Characteristics of *H. pylori* strains and encoded HtrA proteins used in this study[a].

H. pylori strain	Origin	Pathology	*htrA* gene size (bp)	HtrA protein size (aa)	Accession Number	Reference	HtrA secretion[b]	E-cadherin cleavage[c]	HtrA mutant[d]
26695	UK	Gastritis	1,428	476	AFV42231.1	Tomb *et al.*, 1997	+	+	-
Lit75	Lithuania	Non-atrophic gastritis	1,428	476	ADU82875.1	Kersulyte *et al.*, 2010	+	+	-
Sat464	Peru	Not evaluated directly [Non-atrophic gastritis assumed]	1,428	476	ADO05177.1	Kersulyte *et al.*, 2010	+	+	-
Gam94/24	Gambia	Not evaluated directly [Non-atrophic gastritis assumed]	1,428	476	ADU81307.1	Kersulyte *et al.*, 2010	+	+	-
Safr7	South Africa	Not evaluated directly [Non-atrophic gastritis assumed]	1,428	476	ADU84449.1	Duncan *et al.*, 2013	+	+	-
B128	USA	Peptic ulcer	1,428	476	EEC25455.1	McClain *et al.*, 2009	+	+	-
B8	USA	Peptic ulcer	1,428	476	CBI66691.1	Farnbacher *et al.*, 2010	+	+	-
J99	USA	Duodenal ulcer	1,428	476	AAD05980.1	Alm *et al.*, 1998	+	+	-
G27	Italy	n.s.	1,428	476	ACI27174.1	Baltrus *et al.*, 2009	+	+	-
HPAG1	Sweden	Atrophic gastritis	1,428	476	ABF84495.1	Oh *et al.*, 2006	+	+	-
TN2-GF4	Japan	Gastric ulcer	1,428	476	---	unpublished	+	+	-
83	Japan	Gastric cancer	1,428	476	AEE70094.1	Yahara *et al.*, 2013	+	+	-
35A	Japan	Gastric ulcer	1,428	476	ADU40801.1	Yahara K *et al.*, 2013	+	+	-
Shi470	Peru	Not evaluated directly [Non-atrophic gastritis assumed]	1,428	476	ACD47894.1	Kersulyte *et al.*, 2010	+	+	-
Cuz20	Peru	Not evaluated directly [Non-atrophic gastritis assumed]	1,428	476	ADO03669.1	Kersulyte *et al.*, 2010	+	+	-
Pecan4	Peru	Gastric cancer	1,428	476	ADO06675.1	Kersulyte *et al.*, 2010	+	+	-
P12	Germany	Duodenal ulcer	1,428	476	ACJ07582.1	Fischer *et al.*, 2010	+	+	-
SJM180	Peru	Gastritis	1,428	476	ADO02048.1	Kersulyte *et al.*, 2010	+	+	-
India7	India	Not evaluated directly [Non-atrophic gastritis assumed]	1,428	476	ADU79653.1	Kersulyte *et al.*, 2010	+	+	-
B38	France	MALT lymphoma	1,428	476	CAX28976.1	Thiberge *et al.*, 2010	+	+	-
SS1	Australia	Peptic ulcer	1,428	476	---	unpublished	+	+	-

[a] Abbreviations used: aa (amino acids), bp (base pairs), n.s. not specified

[b] HtrA secretion in the culture supernatant was done in BHI medium for 12h and tested by Western blotting as described in the Materials and Methods section.

[c] HtrA cleavage of recombinant E-cadherin was done as described in the Materials and Methods section.

[d] HtrA gene knockout mutagenesis was attempted both by natural transformation and electroporation as described in the Materials and Methods section.

Suppl. Figure 1

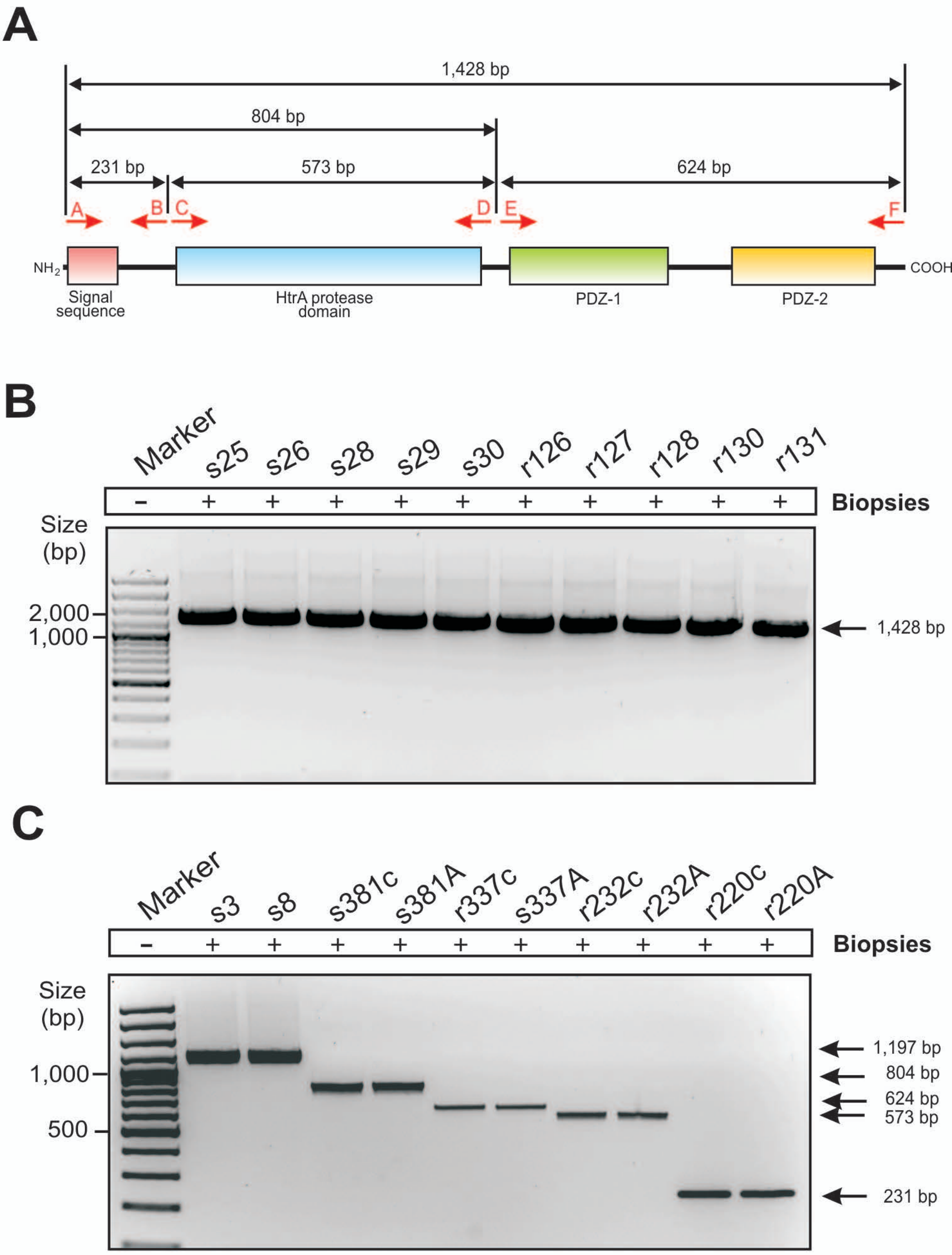

Suppl. Figure 2

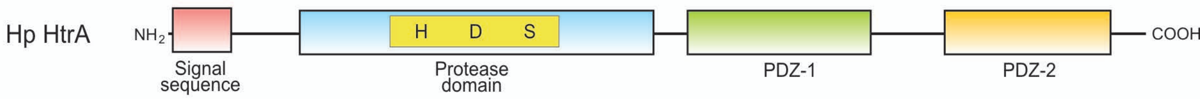


```
South Africa  SouthAfrica7  MVKKTLFISLALALSLNAGNIQIQSMPKVKERISVPSKDDTIYSYHDSIKDSIKAVVNIS
Peru          Cuz20         MMKKTLFISLALALSLNAGNIQIQSMPKVKERVSVPSKDDTIYSYHDSIKDSIKAVVNIS
India         India7        MMKKTLFISLALALSLNAGNIQIQNMPKVKERVSVPSKDDTIYSYHDSIKDSIKAVVNIS
Japan         TN2-GF4       MMKKTLFISLALALSLNAGNIQIQSMPKVKERVSVPSKDDTIYSYHDSIKDSIKAVVNIS
Gambia        Gambia94/24   MMKKTLFISLALALSLNAGNIQIQNMPKVKERISVPSKEDTIYSYHDSIKDSIKAVVNIS
Sweden        HPAG1         MMKKTLFISLALALSLNAGNIQIQNMPKVKERVSVPSKDDTIYSYHDSIKDSIKAVVNIS
USA           B128          MMKKTLFISLALALSLNAGNIQIQNMPKVKERVSVPSKDDTIYSYHDSIKDSIKAVVNIS
                            *:**********************.*******:*****:*********************

              SouthAfrica7  TEKKIKNNFMGSGMFNDPFFQQFFGDLGGMIPKERMERALGSGVIISKDGYIVTNNHVID
              Cuz20         TEKKIKNSFIGGGVFNDPFFQQFFGDLGGMIPKERMERALGSGVIISKDGYIVTNNHVID
              India7        TEKKIKNNFIGGGVFNDPFFQQFFGDLGGMIPKERMERALGSGVIISKDGYIVTNNHVID
              TN2-GF4       TEKKIKNNFIGGGVFNDPFFQQFFGDLGGMIPKERMERALGSGVIISKDGYIVTNNHVID
              Gambia94/24   TEKKIKNNFIGGGVFNDPFFQQFFGDLGGMIPKERMERALGSGVIISKDGYIVTNNHVID
              HPAG1         TEKKIKNNFIGGGVFNDPFFQQFFGDLGGMIPKERMERALGSGVIISKDGYIVTNNHVID
              B128          TEKKIKNNFIGGGVFNDPFFQQFFGDLGGMIPKERMERALGSGVIISKDGYIVTNNHVID
                            *******.*:*.*:**********************************************

              SouthAfrica7  GADKIKVTIPGSNKEYSATLVGTDSESDLAVIRITKDNLPTIKFSDSNDILVGDLVFAIG
              Cuz20         GADKIKVTIPGSNKEYSATLVGTDSESDLAVIRITKDNLPTIKFSDSNDILVGDLVFAIG
              India7        GADKIKVTIPGSNKEYSATLVGTDSESDLAVIRITKDNLPTIKFSDSNDILVGDLVFAIG
              TN2-GF4       GADKIKVTIPGSNKEYSATLVGTDSESDLAVIRITKDNLPTIKFSDSNDILVGDLVFAIG
              Gambia94/24   GADKIKVTIPGSNKEYSATLVGTDSESDLAVIRITKDNLPTIKFSDSNDILVGDLVFAIG
              HPAG1         GADKIKVTIPGSNKEYSATLVGTDSESDLAVIRITKDNLPTIKFSDSNDISVGDLVFAIG
              B128          GADKIKVTIPGSNKEYSATLVGTDSESDLAVIRITKDNLPTIKFSDSNDISVGDLVFAIG
                            ************************************************** *********

              SouthAfrica7  NPFGVGESVTQGIVSALNKSGIGINSYENFIQTDASINPGNSGGALIDSRGGLVGINTAI
              Cuz20         NPFGVGESVTQGIVSALNKSGIGINSYENFIQTDASINPGNSGGALIDSRGGLVGINTAI
              India7        NPFGVGESVTQGIVSALNKSGIGINSYENFIQTDASINPGNSGGALIDSRGGLVGINTAI
              TN2-GF4       NPFGVGESVTQGIVSALNKSGIGINSYENFIQTDASINPGNSGGALIDSRGGLVGINTAI
              Gambia94/24   NPFGVGESVTQGIVSALNKSGIGINSYENFIQTDASINPGNSGGALIDSRGGLVGINTAI
              HPAG1         NPFGVGESVTQGIVSALNKSGIGINSYENFIQTDASINPGNSGGALIDSRGGLVGINTAI
              B128          NPFGVGESVTQGIVSALNKSGIGINSYENFIQTDASINPGNSGGALIDSRGGLVGINTAI
                            ************************************************************

              SouthAfrica7  ISKTGGNHGIGFAIPSNMVRDIVTQLIKTGKIERGYLGVGLQDVSGDLQNSYDNKEGAVV
              Cuz20         ISKTGGNHGIGFAIPSNMVKDIVTQLIKTGKIERGYLGVGLQDLSGDLQNSYDNKEGAVV
              India7        ISKTGGNHGIGFAIPSNMVKDIVTQLIKTGKIERGYLGVGLQDLSGDLQNSYDNREGAVV
              TN2-GF4       ISKTGGNHGIGFAIPSNMVKDIVTQLIKTGKIERGYLGVGLQDLSGDLQNSYDNKEGAVV
              Gambia94/24   ISKTGGNHGIGFAIPSNMVKDIVTQLIKTGKIERGYLGVGLQDLSGDLQNSYDNKEGAVV
              HPAG1         ISKTGGNHGIGFAIPSNMVKDIVTQLIKTGKIERGYLGVGLQDLSGDLQNSYDNKEGAVV
              B128          ISKTGGNHGIGFAIPSNMVKDIVTQLIKTGKIERGYLGVGLQDLSGDLQNSYDNKEGAVV
                            *******************:***********************:**********:*****

              SouthAfrica7  ISVEKDSPAKKAGILVWDLITEVNGKRVKNSNELRNLIGSMLPNQRVTLKVIRDKKERVF
              Cuz20         ISVEKDSPAKKAGILVWDLITEVNGKKVKNTNELRNLIGSMLPNQRVTLKVIRDKKERAF
              India7        ISVEKDSPAKKAGILVWDLITEVNGKKVKNTNELRNLIGSMLPNQRVTLKVIRDKKERAF
              TN2-GF4       ISVEKDSPAKKAGILVWDLITEVNGKKVKNTNELRNLIGSMLPNQKVTLKVIRDKKERTF
              Gambia94/24   ISVEKDSPAKKAGILVWDLITEVNGKKVKNTNELRNLIGSMLPNQRVTLKVIRDKKERTF
              HPAG1         ISVEKDSPAKKAGILVWDLITEVNGKKVKNTNELRNLIGSMLPNQRVTLKVIRDKKERTF
              B128          ISVEKDSPAKKAGILVWDLITEVNGKKVKNTNELRNLIGSMLPNQRVTLKVIRDKKERTF
                            **************************:***:**************:************.*

              SouthAfrica7  TLTLAERKNPNKKETISSQNGAQGQLNGLQVEDLTQKTKKSMRLSDDVQGVLVSQVNENS
              Cuz20         TLTLAERKNPNKKETISAQNGAQGQLNGLQVEDLTQKTKRSMRLSDDVQGVLVSQVNENS
              India7        TLTLAERKNPNKKETISAQNGSQGQLNGLQVEDLTQKTKRSMRLSDDVQGVLVSQVNENS
              TN2-GF4       TLTLAERKNPNKKETISAQNGAQGQLNGLQVEDLTQKTKRSMRLSDDVQGVLVSQVNENS
              Gambia94/24   TLTLAERKNPNKKETISAQNGAQGQLNGLQVEDLTQKTKRSMRLSDDVQGVLVSQVNENS
              HPAG1         TLTLAERKNPNKKETISAQNGAQGQLNGLQVEDLTQKTKRSMRLSDDVQGVLVSQVNENS
              B128          TLTLAERKNPNKKETISAQNGAQGQLNGLQVEDLTQKTKRSMRLSDDVQGVLVSQVNENS
                            *****************:***:*****************:********************

              SouthAfrica7  PAGQAGFRQGNIITRIEDIEIKSVTDFNHALEKYKGKPKRFLVLDLNQGYRLILVK
              Cuz20         PAEQAGFRQGNIITKIEEVEVKSVADFNHALEKYKGKPKRFLVLDLNQGYRIILVK
              India7        PAEQAGFRQGNIITKIEEIEVKSVADFNHALEKYKGKPKRFLVLDLNQGYRIILVK
              TN2-GF4       PAEQAGFRQGNIITKIEEIEVKSVADFNHALEKYKGKPKRFLVLDLNQGYRIILVK
              Gambia94/24   PAEQAGFRQGNIITKIEEIEVKSVADFNHALEKYKGKPKRFLVLDLNQGYRIILVK
              HPAG1         PAEQAGFRQGNIITKIEEIEVKSVADFNHALEKYKGKPKRFLVLDLNQGYRIILVK
              B128          PAEQAGFRQGNIITKIEEIEVKSVADFNHALEKYKGKPKRFLVLDLNQGYRIILVK
                            ** ***********:**::*:***:**************************:****
```

Suppl. Figure 3

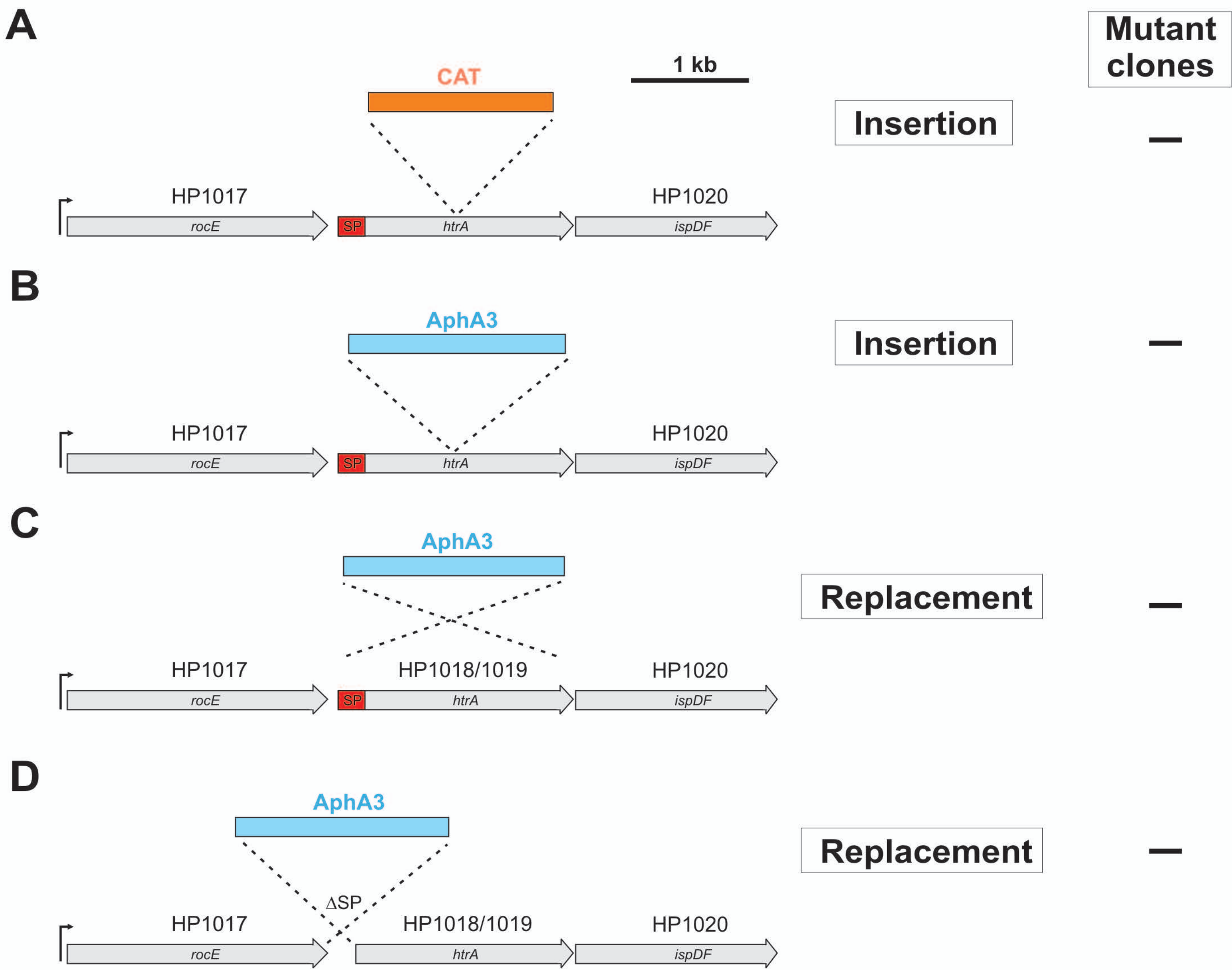

Suppl. Figure 4

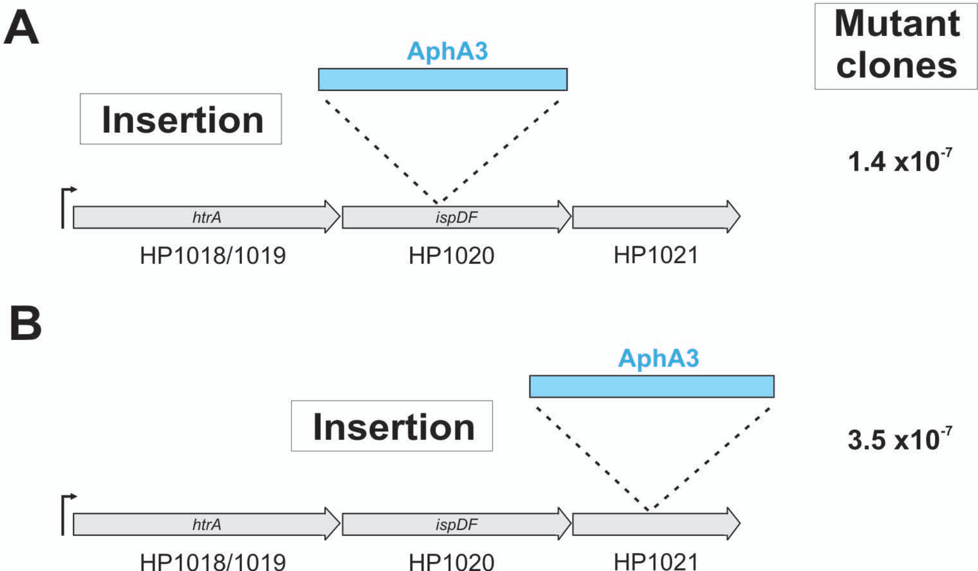

Suppl. Figure 5

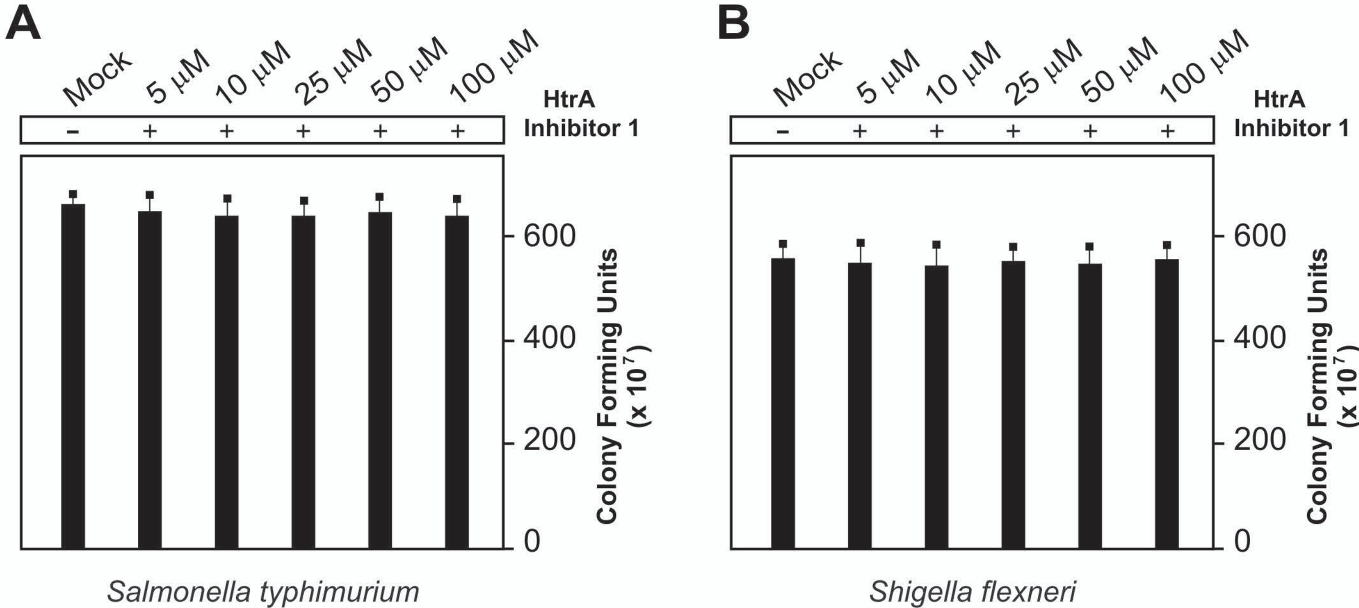

Supplement: Supplementary file 1 — Supporting information [file MMI-99-925-s001.pdf]
